# Loss of Dystroglycan Drives Cellular Senescence via Defective Mitosis-Mediated Genomic Instability

**DOI:** 10.3390/ijms21144961

**Published:** 2020-07-14

**Authors:** Guadalupe Elizabeth Jimenez-Gutierrez, Ricardo Mondragon-Gonzalez, Luz Adriana Soto-Ponce, Wendy Lilián Gómez-Monsiváis, Ian García-Aguirre, Ruth Abigail Pacheco-Rivera, Rocío Suárez-Sánchez, Andrea Brancaccio, Jonathan Javier Magaña, Rita C.R. Perlingeiro, Bulmaro Cisneros

**Affiliations:** 1 Departamento de Genética y Biología Molecular, Centro de Investigación y de Estudios Avanzados del Instituto Politécnico Nacional, Ciudad de México 07360, Mexico; gjimenezg@cinvestav.mx (G.E.J.-G.); rmondragon90@gmail.com (R.M.-G.); luz.ponce@cinvestav.mx (L.A.S.-P.); wlgomez@cinvestav.mx (W.L.G.-M.); ian.garcia@cinvestav.mx (I.G.-A.); 2 Departamento de Bioquímica, Escuela Nacional de Ciencias Biológicas, Instituto Politécnico Nacional, Ciudad de México 11340, Mexico; rpachecor@ipn.mx; 3 Departamento de Genética, Laboratorio de Medicina Genómica, Instituto Nacional de Rehabilitación “Luis Guillermo Ibarra Ibarra”, Ciudad de México 14389, Mexico; srossmary@gmail.com; 4 School of Biochemistry, University of Bristol, Bristol BS8 1TD, UK; andrea.brancaccio@icrm.cnr.it; 5 Institute of Chemical Sciences and Technologies “Giulio Natta” (SCITEC), 00168 Roma, Italy; 6 Departamento de Bioingeniería, Escuela de Ingeniería y Ciencias, Instituto Tecnológico y de Estudios Superiores de Monterrey-Campus Ciudad de México, Ciudad de México 14380, Mexico; 7 Department of Medicine, Lillehei Heart Institute, University of Minnesota, Minneapolis, MN 55455, USA; perli032@umn.edu

**Keywords:** β-Dystroglycan, cellular senescence, lamin B1, DNA-damage response, defective mitosis

## Abstract

Nuclear β-dystroglycan (β-DG) is involved in the maintenance of nuclear architecture and function. Nonetheless, its relevance in defined nuclear processes remains to be determined. In this study we generated a C2C12 cell-based DG-null model using CRISPR-Cas9 technology to provide insights into the role of β-DG on nuclear processes. Since DG-null cells exhibited decreased levels of lamin B1, we aimed to elucidate the contribution of DG to senescence, owing to the central role of lamin B1 in this pathway. Remarkably, the lack of DG enables C2C12 cells to acquire senescent features, including cell-cycle arrest, increased senescence-associated-β-galactosidase activity, heterochromatin loss, aberrant nuclear morphology and nucleolar disruption. We demonstrated that genomic instability is one driving cause of the senescent phenotype in DG-null cells via the activation of a DNA-damage response associated with mitotic failure, as shown by the presence of multipolar mitotic spindles, which in turn induced the formation of micronuclei and γH2AX foci (DNA-damage marker), telomere shortening and p53/p21 upregulation. Altogether, these events might ultimately lead to premature senescence, impeding the replication of the damaged genome. In summary, we present evidence supporting a role for DG in protecting against senescence, through the maintenance of proper lamin B1 expression/localization and proper mitotic spindle organization.

## 1. Introduction

Dystroglycan (DG) is an integral membrane complex that connects the extracellular matrix (ECM) with the intracellular actin-based cytoskeleton, providing structural stability to the plasma membrane (PM) in different tissues and cell types [1,2,3]. DG is synthesized as a propeptide that separates into α- and β-DG subunits after proteolytic cleavage [2,4,5]. Both subunits remain together at the PM through the interaction between β-DG’s extracellular domain and α-DG’s carboxy-terminal globular domain. While α-DG is an extracellular peripheral glycoprotein that binds to various extracellular matrix molecules, including laminin, agrin and perlecan, β-DG is a single-pass transmembrane protein that binds through its cytoplasmic tail to dystrophin, caveolin-3 and other cytoplasmic proteins involved in signal transduction [6,7,8,9,10]. Perturbation of dystroglycan processing is associated with severe congenital disorders and cancer progression [11,12]. In addition, DG has been implicated in cellular processes such as signal transduction and tissue morphogenesis. DG is particularly relevant in skeletal muscle tissue, where it has been classically described to play a key role in stabilizing the sarcolemma of myofibers during the cycles of muscle contraction and relaxation [13]. Upon injury, muscle-specific stem cells (i.e., satellite cells) are activated, proliferate and differentiate into myoblasts that can fuse with pre-existing myofibers, or form new fibers, to overcome the muscle damage. Interestingly, DG is also expressed in satellite cells, where it is essential to enable skeletal muscle regeneration [13,14]. In myoblasts, DG plays an important role in modulating myoblast motility and migration [15]. Therefore, DG has been proven relevant not only in myofibers, but also in myogenic precursors for proper muscle function.

Interestingly, β-DG has the ability to traffic from PM to the nucleus, using the membranous endosome–endoplasmic reticulum network and the importin α2/β1 nuclear import pathway [16,17,18]. This additional cellular localization suggests potential further roles for β-DG. For instance, nuclear β-DG has been involved in the transcriptional regulation of androgen-responsive transcription factors in prostate cancer [19]. We previously demonstrated that β-DG assembles with the nuclear envelope (NE) proteins emerin and lamins A/C and B1 to maintain nuclear architecture and function in myoblasts [20]. β-DG is subject to nucleocytoplasmic shuttling with an active exportin1/CRM1-mediated nuclear export pathway [21] that together with its nuclear import serves to tightly regulate the nuclear levels of β-DG, thereby allowing effective interactions with binding partners at the NE interface. However, the molecular basis underlying the role of β-DG on NE-associated functions is largely unknown. In this study, we generated DG-null mouse myoblasts (C2C12) using CRISPR-Cas9 technology to analyze in depth the function of β-DG in the nucleus. The initial phenotype noted in DG-null cells was the decrease in lamin B1 levels, which was accompanied by nuclear morphology defects. Therefore, taking the evidence that lamin B1 plays a pivotal role in cellular senescence [22,23,24,25], we analyzed the contribution of DG to this cellular process. Cellular senescence is defined as a state of permanent cell cycle arrest that occurs in response to different damaging stimuli, including persistent DNA damage, telomere shortening, oxidative stress and oncogenic signaling [26,27,28], with silencing of lamin B1 expression being an early and necessary event for senescence to be established [22,23,24,25]. We provided evidence showing that DG plays a protective role against senescence, because the lack of DG makes C2C12 cells to acquire senescent features. In addition, we demonstrated that senescence signaling in DG-null cells is triggered by mitotic failure, which in turn elicits a p53-mediated DNA-damage response to arrest the cell cycle, leading to premature senescence.

## 2. Results

### 2.1. Generation and Characterization of CRISPR/Cas9-Mediated DG-Null C2C12 Cell Clones

**Figure 1:**
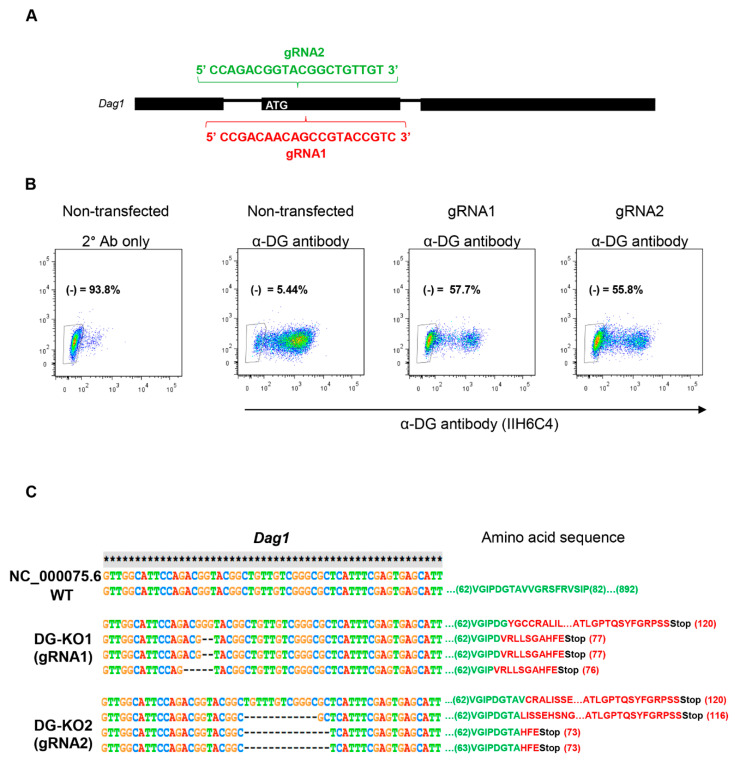
CRISPR/Cas9-engineered dystroglycan knockout (DG-KO) C2C12 cell clones. (**A**) Scheme showing the sequences of guide RNAs (gRNA1 and gRNA2), designed to target *Dag1* gene. (**B**) Fluorescence-activated cell sorting (FACS) analysis on C2C12 cells, gRNA2 or none gRNA (non-transfected cells), and stained with α-DG antibody, IIH6C4. The absence of IIH6C4 reactivity confirmed the lack of functionally glycosylated α-DG. Non-transfected cells incubated only with secondary antibody (2° Ab only) were used to adjust the population negative for α-DG immunostaining (α-DG (-)). Percentages correspond to α-DG (-) population. (**C**) Sequence alignment of mouse *Dag1* gene (annotated) showing the introduction of indels in DG-KO1 and DG-KO2 cell lines compared with WT cells. Amino acid sequence shows the position of the stop codons generated in DG-KO1 and DG-KO2 cell clones.

To analyze in depth the functional relationship of β-DG with the NE, we engineered DG knockout cells (DG-KO) on the mouse myogenic cell line C2C12, using CRISPR/Cas9. To silence DG expression, C2C12 cells were transfected with a vector expressing Cas9, the red fluorescence protein (RFP) and one of two different guide RNAs (gRNA1 and gRNA2) targeting the region downstream of the ATG translation initiation codon within the first coding exon of the mouse *Dag1* gene (Figure 1A). After positive selection for RFP and two rounds of negative selection using the IIH6 antibody, which is specific to the αDG laminin binding domain [1,29] fluorescence-activated cell sorting (FACS) and further clonal expansion, two different KO lines (DG-KO1 and DG-KO2) were selected (Figure 1B; see Methods for details). DNA sequencing of the target site was performed to directly identify editing events. Both DG-KO clones showed indels that generate premature stop codons; thus, only polypeptides with presumably no biological activity are synthesized from DG-KO clones (Figure 1C).

**Figure 2:**
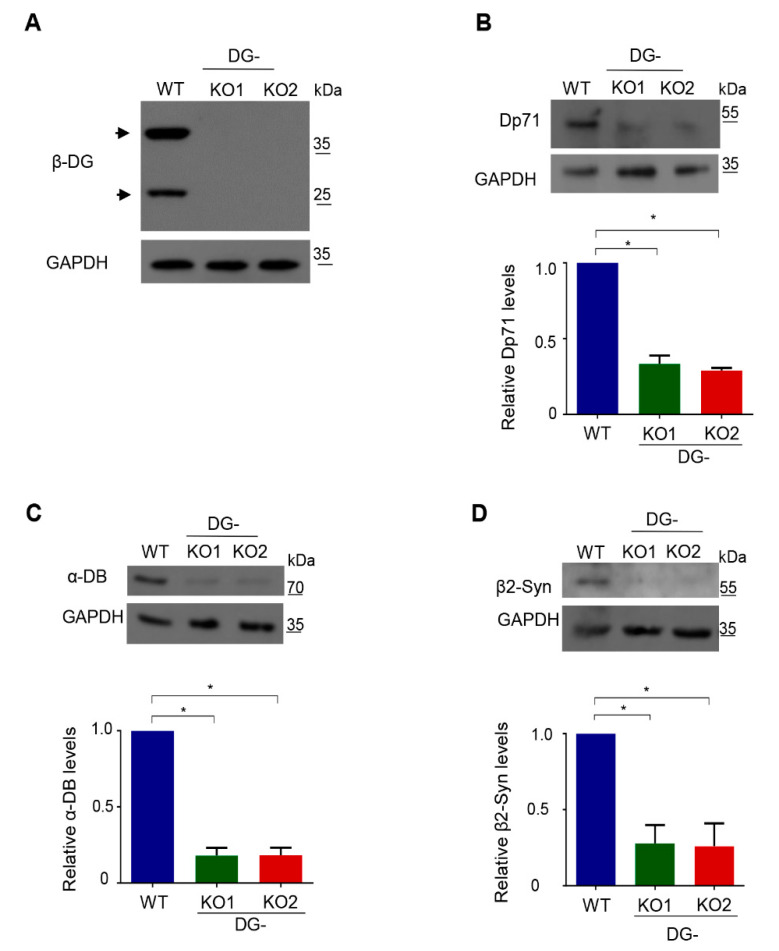
Decreased protein levels of dystrophin associated proteins in DG-KO cells. Lysates from WT, DG-KO1 and DG-KO2 cell cultures were analyzed by SDS-PAGE/WB using specific antibodies against β-DG (**A**), Dp71 (**B**), α-dystrobrevin (α-DB) (**C**), β2-syntrophin (β2-Syn) (**D**) and GAPDH (loading control); representative blots are shown. Bottom graphs: relative protein expression was calculated from three independent experiments and significant differences were calculated using one-way ANOVA and Dunnett´s post hoc test; * *p* < 0.05 in comparison to WT. Data indicate the mean ± SEM.

Owing to the functional relationship of DG with dystrophin-associated proteins (DAPs), DG-KO clones were initially characterized by analyzing the protein levels of various DAPs, namely dystrophin Dp71, α-dystrobrevin and β2-syntrophin. Lysates from both DG-KO1 and DG-KO2 clones showed no β-DG protein expression (Figure 2A; 43 kDa and 26 kDa proteins), and a drastic decrease in the levels of all DAPs analyzed was observed, compared with WT cells (Figure 2B–D). Overall, these data validate DG-KO clones as model for studying DG, including the role of β-DG in NE-associated processes.

### 2.2. DG Deficiency Provokes Altered Localization and Decreased Protein Levels of Lamin B1

**Figure 3:**
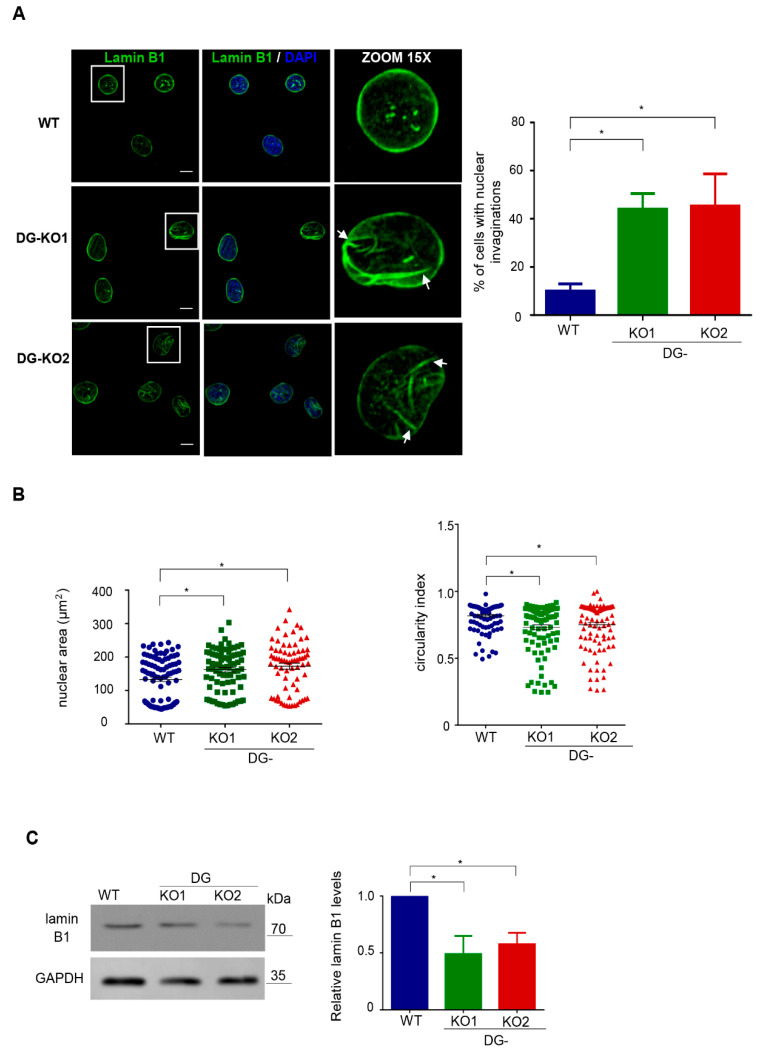
DG-KO cells show altered localization and decreased protein levels of lamin B1. (**A**) WT, DG-KO1 and DG-KO2 cells, seeded on glass coverslips, were fixed and immunostained for lamin B1. Nuclei were stained with diamidino-2-phenylindole (DAPI) prior to confocal laser scanning microscopy analysis (CLSM), and typical images are shown. Bar, 10 µm. Right: the bar graph shows the percentage of nuclei with invaginations, with significant differences calculated in each cell culture from three independent experiments using one-way ANOVA and Dunnett´s post hoc test; data indicate the mean ± SEM (*n* = 100 nuclei per cell culture; * *p* < 0.05 compared to WT). (**B**) Nuclear morphometric analysis was carried out on WT, DG-KO1 and DG-KO2 cells, as described in Methods from three separate experiments; significant differences were obtained using a non-parametric Kruskal–Wallis test and post hoc Dunn´s method. Data correspond to the mean ± SEM (*n* = 100 nuclei per experimental condition; * *p* < 0.05 compared to WT). (**C**) Lysates from WT and DG-KO1 and DG-KO2 cells were analyzed by SDS-PAGE/WB using antibodies against lamin B1 and GAPDH (loading control). Representative blots from three separate experiments are shown, with significant differences obtained using one-way ANOVA and Dunnett´s post hoc test; * *p* < 0.05 in comparison to WT. Data indicate the mean ± SEM. Right: densitometric analysis of immunoblot autoradiograms was performed to estimate lamin B1 protein expression.

Because lamin B1, a critical NE protein, is a β-DG-interacting partner [20], we were prompted to evaluate the impact of the lack of DG on lamin B1 distribution and protein expression. Interestingly, altered immunostaining for lamin B1 and evident nuclear deformities (invaginations) were found in DG-KO1 and DG-KO2 cells (Figure 3A). Consistently, the percentage of cells with aberrant nuclear morphology was clearly higher in DG-KO cell cultures than WT cell culture (right graph). Morphometric analysis of nuclei (nuclear area and circularly index) confirmed significant differences in nuclear shape between WT and DG-KO1 and DG-KO2 cells (Figure 3B). In line with IF/confocal microscopy images, a significant decrease in lamin B1 levels was observed in DG-KO1 and DG-KO2 cells (Figure 3C).

### 2.3. The Loss of DG Induces the Expression of Senescence-Associated Features

**Figure 4:**
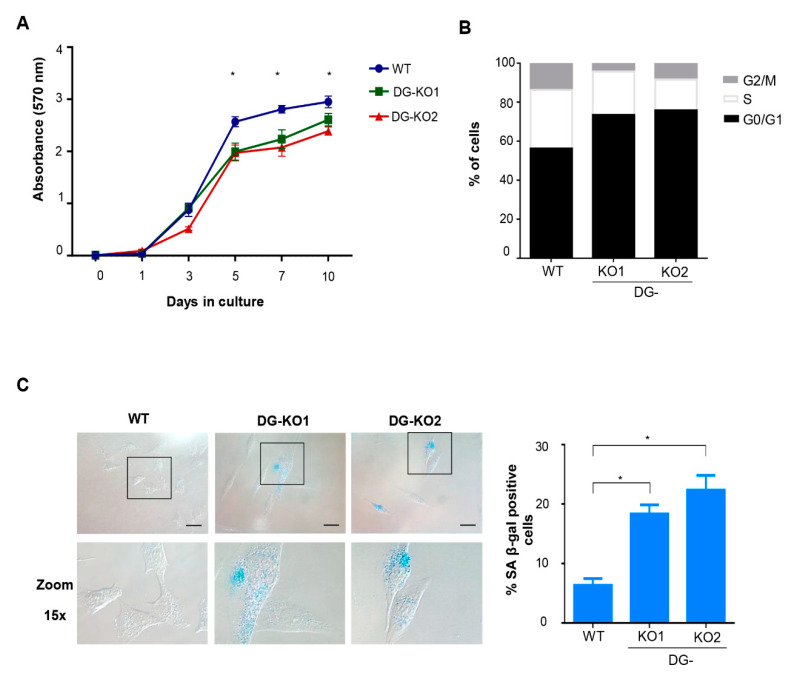
DG-KO cell cultures exhibit decreased proliferation, G0/G1 arrest and senescence. (**A**) MTT-based cell proliferation assays were performed over a 10 days period in WT, DG-KO1 and DG-KO2 cell cultures. Data correspond to the mean ± SEM from three independent experiments, with significant differences determined by one-way ANOVA; * *p* < 0.05 compared to WT. (**B**) Cell cycle analysis on WT, DG-KO1 and DG-KO2 asynchronous cell cultures was performed by flow cytometry. A typical graph from three independent experiments is shown. (**C**) Senescent cells were identified in WT, DG-KO1 and DG-KO2 cultures by quantifying SA β-gal activity, and representative images were acquired by light-field microscopy. Bar = 50 µM. Right: the percentage of senescent cells was calculated, and significant differences were obtained from three separate experiments using one-way ANOVA, followed by Dunnett´s post hoc test. Data correspond to the mean ± SEM (*n* = 200 cells for each cell culture; * *p* < 0.05 compared to WT).

**Figure 5:**
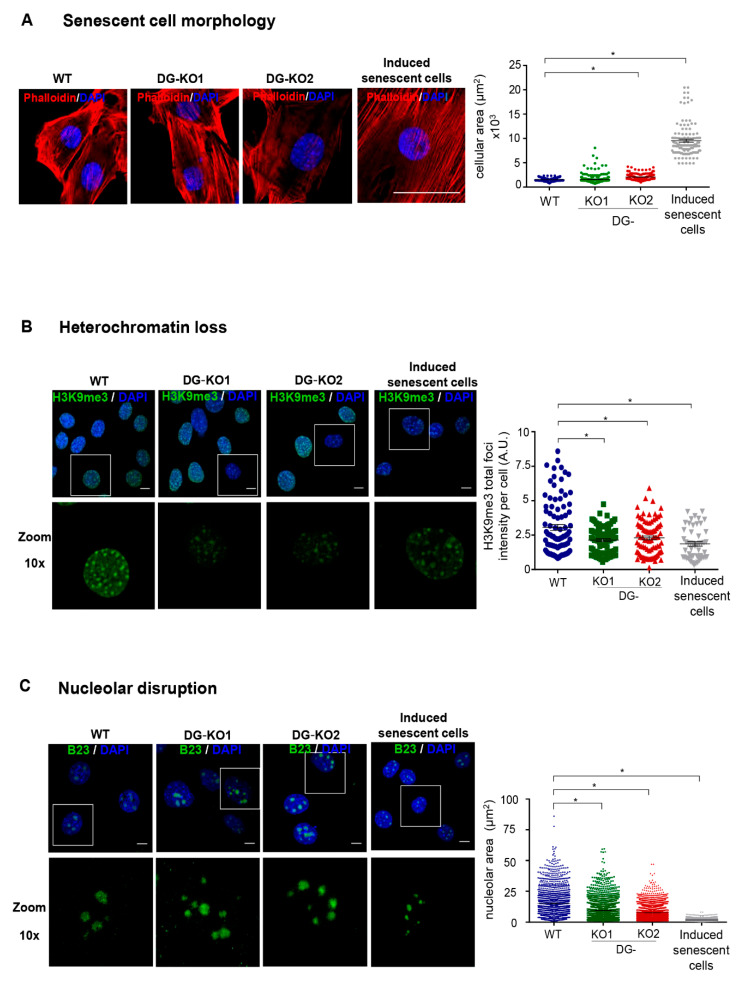
DG-null cells display senescent characteristics. WT, DG-KO1 and DG-KO2 cells were seeded on glass coverslips and fixed prior to CLSM analysis. (**A**) Senescent cell morphology. Cells were labeled with DAPI and phalloidin to visualize nuclei and actin-based cytoskeleton, respectively, and representative images are shown. Bar, 50 µM. Right: the cellular area was estimated using ImageJ software, with significant differences determined by non-parametric Kruskal–Wallis tests and post hoc Dunn´s method. Data correspond to the mean ± SEM (*n* = 200 cells for each cell culture and from three independent experiments). * *p* < 0.05 in comparison to WT). (**B**) Heterochromatin loss. Cell preparations were immunostained for H3K9me3 followed by DAPI labeling to enable nuclei visualization. Representative confocal microscope images are shown (bar, 10 µM). Right: the fluorescent intensity of H3K9me3 foci was measured using ImageJ software, as described in Methods. Significant differences were determined by non-parametric Kruskal–Wallis tests, followed by post hoc Dunn´s method. Data correspond to the the mean ± SEM (*n* = 200 cells for each cell culture and from three independent experiments; * *p* < 0.05 in comparison to WT). (**C**) Nucleolar disruption. Cell preparations were immunostained for B23 and labeled with DAPI to decorate nucleoli and nuclei, respectively. Scale bar, 10 µM. Right: nucleolar area was assessed using ImageJ software, as described in Methods (*n* = 1300 nucleoli per experimental condition). Significant differences were determined by non-parametric Kruskal–Wallis tests and post hoc Dunn´s method; data indicate the mean ± SEM.; * *p* < 0.05 compared to WT.

Lamin B1 downregulation occurs at the onset of cell transition to senescence [23,30]. Thus, we tested whether the reduction in lamin B1 as a consequence of DG loss would lead to a senescent phenotype. To approach this idea, we searched for senescence characteristics in DG-null cells. Decreased proliferative potential and a higher percentage of cells at the G0/G1 phase of the cell cycle were found in DG-KO1 and DG-KO2 cell cultures, compared with WT cells, as shown by MTT and flow cytometry analyses, respectively (Figure 4A,B). G0/G1 arrest might be indicative of premature senescence; thus, the identification of senescent cells in DG-KO cultures was carried out using senescence-associated β-galactosidase activity [SA-β-gal]. Interestingly, increased numbers of senescent cells were found in DG-KO1 and DG-KO2 cultures (20–25%), compared with WT cells (5%) (Figure 4C). In addition to increased β-galactosidase activity, senescent cells display large-flat cell morphology and undergo both nucleolar stress and the loss of perinuclear chromatin [28], among other characteristics. To corroborate the increase in senescent cells in DG-KO cultures, we searched for the presence of these features in DG-null cell cultures, using WT cells induced to senescence by treatment with sodium butyrate (NaBu) for ten days as a positive control for the senescent phenotype. NaBu is a histone deacetylase inhibitor that elicits senescence via irreversible induction of cell cycle arrest [31]. WT cells treated with NaBu showed a flattened and expanded morphology, which markedly contrasts with the typical polygonal morphology of untreated WT cells (Figure 5A). DG-KO2 but not DG-KO1 cells showed a subtle but statistically significant increase in cell surface, compared with WT cells, as determined by phalloidin staining of F-actin and estimation of cell surface area (Figure 5A and right graph). On the other hand, a marked decrease in H3K9me3 foci immunostaining (heterochromatin marker) was observed in DG-null cells in a similar fashion to that observed in NaBu-induced senescent cells (Figure 5B), as determined by confocal laser scanning microscopy (CLSM) and fluorescence intensity quantification (Figure 5B and right graph). Finally, disaggregated nucleoli with a smaller area were found in DG-KO1 and DG-KO2 cell cultures, compared with WT cells, as revealed by immunostaining for the nucleolar protein B23 and nucleolar area quantification (Figure 5C and right graph). However, nucleolar disaggregation was much greater in NaBu-induced senescent cells.

**Figure 6:**
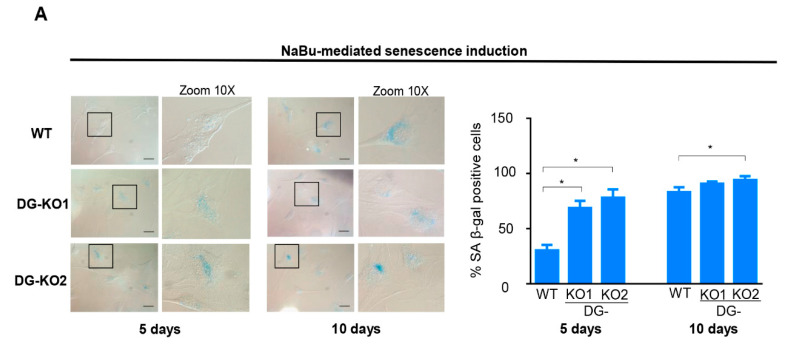
The loss of DG makes C2C12 cells more responsive to senescence induction. (**A**)WT, DG-KO1, and DG-KO2 cell cultures were treated with sodium butyrate (NaBu) for 5 or 10 days to induce senescence, and senescent cells were identified by SA β-gal activity; typical images were acquired by light-field microscopy. Bar = 50 µM. Right: graph shows the percentage of senescent cells obtained from three independent assays (*n* = 100 cells for each cell culture). Significant differences were determined by one-way ANOVA followed by Dunnett´s multiple comparison test; * *p* < 0.05 compared to WT. Data correspond to the mean ± SEM.

Collectively, the aforementioned data imply that incipient senescence is present in DG-KO cell cultures in the absence of any detectable senescence-inducing stimuli. Thus, we assessed whether the lack of DG sensitizes C2C12 cells to senescence induction. In line with this, the percentage of senescent cells was significantly higher in DG-KO1 cultures (60–70%) than that in WT cells (30%) upon five days of NaBu treatment (Figure 6A). Long-term treatment (10 days) rendered a similar percentage of senescent cells (~75%) between WT and DG-KO cell cultures (Figure 6A).

### 2.4. Aberrant Multipolar Mitoses in DG-KO Cells Resulted in Micronuclei Formation and Activation of a P53-Mediated DNA Damage Response

**Figure 7:**
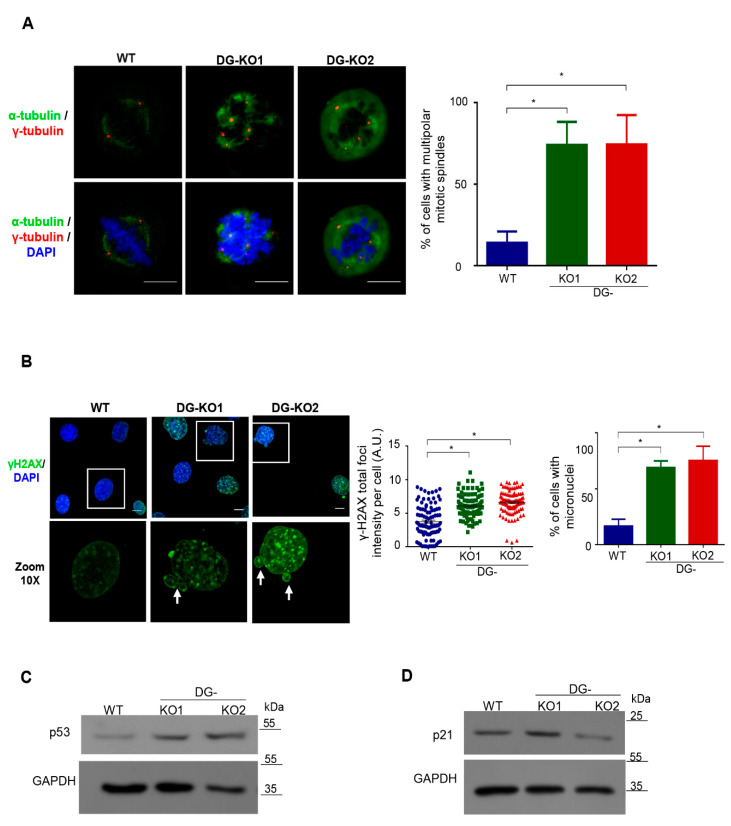
Mitotic failure activates a p53-dependent DNA damage response in DG-null cells. (**A**) WT, DG-KO1 and DG-KO2 cells cultured on coverslips were arrested in S phase by double treatment with thymidine and further release into cell cycle for 4 h to progress into mitosis. Afterwards, cell preparations were immunolabeled for α-tubulin and γ-tubulin to decorate mitotic spindles and centrosomes, respectively, and counterstained with DAPI to visualized nuclei, prior to CLSM analysis. Right. The percentage of multipolar mitotic spindles was determined from three separate experiments (*n* = 50 cells from each cell culture). Significant differences were calculated using one-way ANOVA and Dunnett´s multiple comparison test; * *p* < 0.05 compared to WT. Data indicate the mean ± SEM. (**B**) WT, DG-KO1 and DG-KO2 cells were cultured on coverslips and treated with thymidine as per panel A. Cell preparations were then immunolabeled for γ2HAX and counterstained with DAPI to decorate nuclei, prior to CLSM analysis. Right: the fluorescence intensity of γ2HAX foci was calculated, and significant differences were determined by non-parametric Kruskal–Wallis tests followed by Dunn´s post hoc analysis. Data correspond to the mean ± SEM from three separate experiments; * *p* <0.05 compared to WT. Far right: the number of cells with micronuclei was calculated and significant differences were obtained by one-way ANOVA and Dunnett´s post hoc analyses. Data correspond to the mean ± SEM from three independent assays (*n* = 100 cells for each cell culture); * *p* < 0.05 compared to WT. (**C**) and (**D**) Lysates from WT, DG-KO1 and DG-KO2 cells cultures, previously treated with thymidine as per panel A, were analyzed by SDS-PAGE/WB, using specific antibodies against p53 (C), p21 (D) and the loading control, and representative blots from two independent experiments are shown.

A previous study from our group showed that downregulation of DG in C2C12 cells results in an increased number of centrosomes [20]. Therefore, we next assessed whether the lack of DG would lead to aberrant mitosis and, consequently, genomic instability, contributing to the senescent phenotype. WT, DG-KO1 and DG-KO2 cells previously arrested in S phase by double treatment with thymidine were released to allow their progression into mitosis. Cell were immunolabeled for α-tubulin and γ-tubulin to decorate mitotic spindles and centrosomes, respectively, and mitotic cells were visualized by CLSM. Remarkably, a high percentage of DG-KO1 and DG-KO2 cells (80%) showed multipolar mitotic spindles and multidirectional alignment of chromosomes, compared to WT cells (Figure 7A). Because thymidine treatment evokes a DNA damage response by slowing the progression of replication forks [32], we analyzed whether DG-KO cells are more prone to response to DNA damage than WT cells, by monitoring γ-H2AX foci, a DNA repair marker [33]. In line with our hypothesis, a dramatic increase in fluorescence intensity of γ-H2AX foci was observed in thymidine-treated DG-KO cells (Figure 7B) compared with thymidine-treated WT cells. Furthermore, the presence of micronuclei, another faithful indicator of DNA damage and chromosome instability [34], was frequently observed in DG-KO1 (75%) and DG- KO2 (90%) cell cultures upon thymidine treatment, compared with WT culture (20%). To support the latter result, we searched for micronuclei in the absence of any DNA-damage-inducing agent. A significant increase in the percentage of micronuclei-contained cells was observed in DG-KO1 (8%) and DG-KO2 (10%) cultures, compared with WT culture (4%), as shown by lamin B1 immunostaining (Figure S1). Errors in cell division and persistent DNA damage in DG null cells would lead to the activation of the checkpoint proteins p53 and its target proteins p21, which in turn elicits cell cycle arrest and/or senescence. Consistent with this notion, p53 levels were found to be increased in thymidine-treated DG-KO1 and DG-KO2 cells, while augmented levels of p21 were observed only in DG-KO1 cells, compared with WT cells (Figure 7C). Collectively, these data imply that the lack of DG resulted in aberrant multipolar mitosis, which in turn induces a DNA damage response via p53 activation and ultimately cell-cycle arrest.

**Figure 8:**
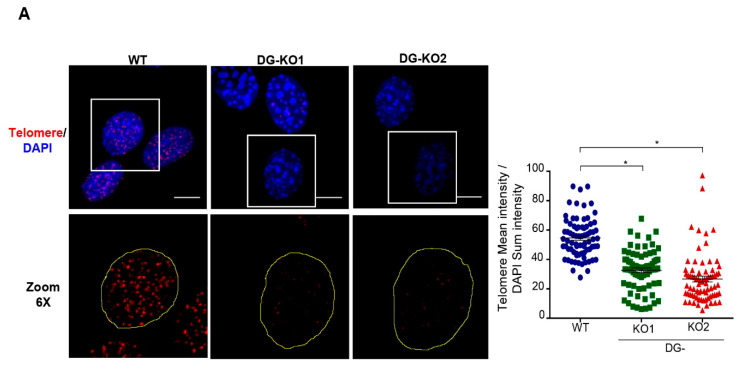
DG-KO cells exhibit telomere shortening. (**A**)WT, DG-KO1 and DG-KO2 cells grown on coverslips were processed for FISH using a specific telomere probe (see Methods), and nuclei were decorated by staining with DAPI. Representative CLSM images are shown; bar, 10 µM. The right graph shows the relative telomere length determined by the telomere mean intensity divided by DAPI sum intensity. Significant differences were determined by non-parametric Kruskal–Wallis tests, followed by post hoc Dunn´s analysis. Data indicate the mean ± SEM from three separate experiments (*n* = 75 cells per cell culture); * *p* < 0.05 compared to WT).

Because DNA damage response can induce telomerase shortening irrespective of telomerase activity [35], we were prompted to estimate telomerase length in DG-deficient and WT cells of similar culture passage (6–8 passage) by in situ hybridization (FISH), using a telomere oligonucleotide fluorescein-labeled probe (Figure 8). It is assumed that the probe hybridizes quantitatively to telomeric repeats, and hence the integrated telomere foci fluorescence intensity of a single nucleus is directly related to the length of their telomeres [36,37]. We observed that the fluorescence intensity of telomere foci was significantly less intense in DG-null cells, compared with WT cells (Figure 8A).

## 3. Discussion

In this study, we generated a C12C12 myoblasts-based model with no expression of DG (α-DG and β-DG) using CRISPR-Cas9 technology to provide insights into the nuclear function of β-DG. In earlier studies, we showed that β-DG is involved in maintaining the structure and function of the NE [20,21]; nevertheless, the specific mechanisms underlying its role in these nuclear processes remains to be determined. We isolated two DG-knockout clones (DG-KO1 and DG-KO2) that were generated from two different gRNAs. We used this strategy in order to validate that the phenotypes observed were not due to off-target cleavage by the CRISPR-Cas9 system. DG-KO cells were intentionally sorted from the glycosylated α-DG negative population and, accordingly, expanded DG-KO clones showed no expression of β-DG and decreased levels of dystrophin Dp71, α-dystrobrevin and β2-syntrophin, three well characterized partners of β-DG [38], which validated our DG-deficient cell system.

We focused our research on the previously observed interaction between β-DG and lamin B1 [20]. Lamin B1 belongs to a group of type V intermediate filament proteins known as the lamins. These proteins are the main component of the nuclear lamina, which provides stability to the nuclear structure, and regulates nuclear processes such as transcription, chromatin organization, cell cycle, among others [39]. The abnormalities in the nuclear lamina observed in DG-KO cells confirmed previous observations suggesting a key role for β-DG in maintaining the integrity of this compartment [21]. Moreover, the decreased levels of lamin B1 in the absence of DG is particularly relevant as the downregulation of lamin B1 is a key event mediating premature senescence [22,40]. This has been attributed to the plethora of nuclear processes that lamin B1 regulates, such as heterochromatin architecture, cell cycle progression, nuclear morphology, gene expression and splicing [41,42]. Therefore, we assessed whether DG-KO cells would acquire a senescent phenotype. Consistently, DG-KO cells exhibited several senescent marks in the absence of any senescence-inducing stimuli, including reduced cell proliferation with arrest at G0/G1, elevated SA-β-gal activity, nucleolar disaggregation, senescent cell morphology and loss of heterochromatin. The cellular transition to senescence is associated with extensive chromatin reorganization and gene expression changes. Specifically, lamin B1 downregulation occurring during senescence facilitates the spatial relocalization of perinuclear H3K9me3-positive heterochromatin [43]. Furthermore, downregulation of SUV39H1 during the establishment of senescence may promote DNA repair, leading to genome destabilization due to deheterochromatinization of repetitive DNA, which in turn results in cell cycle arrest [44]. Thus, lamin B1 may contribute to senescence by the spatial reorganization of chromatin and through gene repression [43]. In this scenery, characterization of the DG-KO cell gene expression profile, including the genomic DNA methylation pattern, will help to determine the epigenetic regulation occurring in response to the loss of DG. It is worth noting that treatment with the histone deacetylase inhibitor NaBu induced senescence in proliferating myoblasts and that this effect was exacerbated in DG-KO cells. A possible explanation is that β-DG is required to stabilize lamin B1 at the nuclear lamina so that it can attenuate induced senescence. Indeed, perturbation of β-DG nuclear trafficking causes both mistargeting and reduced protein levels of lamin B1, leading ultimately to aberrant nuclear architecture [21]. Thus, tight control of nuclear β-DG content is physiologically relevant to preserve β-DG-lamin B1 interaction, thereby allowing the cell to finely tune nuclear activity in response to cellular stimuli. While increased susceptibility to senescence might be a consequence of lamin B1 alteration in the absence of β-DG, it remains to be explored whether additional mechanisms related to β-DG functions (e.g., signaling) are also contributing to this process.

Cellular senescence is induced by different damaging stimuli, including extended replication, DNA damage, oxidative stress, telomere shortening and oncogenic signaling [45,46]. In an attempt to further understand how the lack of DG results in senescence, we invoked an earlier study that might connect DG with DNA damage. We previously demonstrated that DG downregulation resulted in over-duplicated centrosomes in C2C12 cells [20], an aberrant characteristic associated with multipolar mitosis [47,48]. Supporting our assumption, multipolar mitotic spindles were frequently found in DG-null cells, compared with WT culture. Consistent with mitotic defects driving chromosome instability and DNA damage response [49], DG-KO cells exhibited an increased number of micronuclei-containing cells and apparent shortening of telomeres, compared with WT cells. The formation of micronuclei occurred to a much greater extent when DG-null cells were subjected to thymidine-mediated DNA damage. Thymidine treatment evokes a DNA damage response by slowing the progression of replication forks [32]. Furthermore, numerous intensely stained foci of phosphorylated H2AX histone (γH2AX) were found in DG-KO cells after thymidine exposure. γH2AX orchestrates DNA repair by recruiting repair factors to the surrounding of double-strand break (DSB) sites, including MRE11/NBS1/RAD50, MDC1, 53BP1 and BRCA1 [33,50]. Supporting the idea that DG deficiency makes the cell more prone to DNA-damage response, upregulation of the p53 pathway (p53 and p21 proteins) was found in DG-KO cells upon thymidine treatment. p53 plays a pivotal role for senescence induction; the DNA damage response activates ataxia telangiectasia (ATM) and Rad3-related (ATR) kinases, which in turn activate the p53/p21 axis by phosphorylation of both p53 and its ubiquitin ligase Mdm2, leading to the stabilization of p53 levels [51]. However, differences in p53 pathway activation between DG-KO1 and DG-KO2 cells due to inter-clonal heterogeneity cannot be ruled out. This issue deserves further investigation.

How DG-KO cells acquire multiple centrosomes, a hallmark of cancer cells [52], is unknown. Centrosome amplification could result from altered centrosome replication and/or cytokinesis failure. Numerous proteins that regulate the centrosome duplication cycle have been identified, including Polo-like kinase-4, cyclin-dependent kinase 2, and SPD-2 [53]; however, none of them has been linked with DG so far. It is worth noting that β-DG localized to the cleavage furrow and midbody in cytokinesis [54]; thus, DG deficiency might lead centrosome amplification through impaired cytokinesis. Nonetheless, the fact that no binucleated cells were observed in DG-KO cultures opposes this hypothesis. On the other hand, considering that B-type lamins have been involved in the assembly and maintenance of mitotic spindles in Xenopus [55], it is possible that aberrant multipolar spindles in DG-KO cells emerge, at least in part, due to depleted lamin B1 levels exhibited by DG-null cells. Clearly, further research is required to elucidate a role for DG, if any, on centrosome duplication/mitosis organization. Although CRISPR-Cas9 genome editing ablates the expression of both α- and β-DG, we believe that the senescent phenotype of DG-KO cells is mechanistically linked to the nuclear deficiency of β-DG, because lamin B1, the central hub of cellular senescence, is a β-DG interacting partner [20,21]. Nonetheless, the possibility that the lack of α-DG drives the cell to senescence by perturbing the outside-in signaling pathway across the ECM-cytoskeleton-nucleus axis [56] cannot be ruled out. Furthermore, the rescue of DG expression in DG-KO cells is required to undoubtedly demonstrate the contribution of β-DG to cellular senescence.

In summary, overall our data are consistent with the paradigm that interfering with DG function results somehow in aberrant multipolar mitoses, which in turn evokes a p53-dependent DNA-damage response, arresting cell cycle progression and thereby inducing senescence, to avoid propagation of damaged genomes.

## 4. Materials and Methods 

### 4.1. Cell Culturing and Treatments

Mouse C2C12 myoblasts were cultured in Dulbecco’s modified Eagle’s medium (DMEM) (Invitrogen, Carlsbad, CA, USA) supplemented with 10% (*v*/*v*) fetal bovine serum, 50 U/ml penicillin, 50 µg/ml streptomycin, and 1 mM sodium pyruvate at 37 °C, in a humidified 5% CO2 cell incubator. For senescence induction, cells were treated for 5 or 7 days with sodium butyrate (NaBu 5 mM, Sigma-Aldrich, St Louis) diluted in PBS 1X or vehicle alone. To analyze mitosis, cells were blocked at the S phase using double treatment with thymidine (2 mM) and then released from arrest by washing with PBS and plating in fresh culture medium on glass coverslips for 3–4 h (metaphase-anaphase).

### 4.2. Generation of DG-KO C2C12 Cell Lines by CRISPR-Cas 

Two different single guide RNAs were designed to target the first coding exon of *Dag1* gene, using the crispr.mit.edu online tool: gRNA1 (5’ CCGACAACAGCCGTACCGTC 3’) and gRNA2 (5’ CCAGACGGTACGGCTGTTGT 3’) gRNAs were cloned into pSpCas9(BB)-red fluorescent protein (RFP) plasmid (modified from pSpCas9(BB)-2A-GFP, a gift from Feng Zhan–Addgene plasmid # 48138; http://n2t.net/addgene:48138;RRID:Addgene_48138), Addgene (Watertown, MA, USA). C2C12 cells were transfected with gRNA1- or gRNA2-Cas9-RFP plasmids using lipofectamine (LTX) with plus reagent (Thermo Fisher Scientific, Waltham, MA, USA). Forty-eight hours post transfection, RFP positive cells were sorted using FACSAria (BD Biosciences, Woburn, MA, USA). After expansion, cells were collected with enzyme-free cell dissociation buffer (Gibco–Thermo Fisher Scientific, Waltham, MA, USA) and incubated with anti-CD16/CD32 antibody (Mouse BD Fc Block, 2.4G2; BD Biosciences, Woburn, MA, USA) on ice for 5 minutes, and subsequently with anti-α-DG antibody (IIH6C4) on ice for 30 minutes. Following PBS washes, cells were incubated with goat anti-mouse IgG-Alexa Fluor 488 secondary antibody on ice for 20 minutes. Cells were then washed with PBS and resuspended in FACS buffer (10% FBS in PBS). Cells negative for α-DG staining were sorted by FACS and expanded. WT cells incubated with or without IIH6C4 were used to set the gates for positive or negative α-DG staining, respectively. Upon expansion, sorted cells had a second round of sorting for α-DG negative staining and single cells were collected in a 96-well plate for clonal expansion. DG-KO clones were screened for β-DG by Western blotting using anti-β-DG antibodies (MANDAG2). Two clones, DG-KO1 and DG-KO2, were expanded and characterized by sequencing the DNA region targeted by the gRNAs, to confirm *Dag1* gene disruption. Cell cultures between passage six and twelve were used for all analyses.

### 4.3. Antibodies

The following primary antibodies were used. Mouse monoclonal antibodies against α-dystrobrevin (α-DB; BD Transduction Laboratories, Becton Dickinson, Franklin Lakes, NJ, USA), α-DG (IIH6C4 (IIH6, 05-593; Millipore, Sigma-Aldrich, St. Louis, MO, USA), β-DG (MANDAG2 [57]), α-tubulin (sc-32293; Santa Cruz Biotechnology, CA, USA), p53 (#2524; Cell Signaling Technology, MA, USA), p21 (#2946; Cell Signaling Thecnology, MA, USA), and GAPDH (sc-32233; Santa Cruz Biotechnology, CA, USA). Rabbit polyclonal antibodies against B23 (sc-6013-R; Santa Cruz Biotechnology, CA, USA), dystrophin Dp71 (+78Dp71; Genemed Synthesis Inc., San Francisco, CA, USA), lamin B1 (Ab16048; Abcam, Cambridge, UK), ϒ-tubulin (sc10732; Santa Cruz Biotechnology, CA, USA), H3K9me3 (ab8898; Abcam, Cambridge, UK) and γ-H2AX (#07-164; Millipore, Sigma-Aldrich, St. Louis, MO, USA). Goat polyclonal antibody against β2-syntrophin (SC-13766; Santa Cruz Biotechnology, CA, USA) was also used.

### 4.4. Western Blotting 

C2C12 cell culture lysates were electrophoresed on 10% SDS-polyacrylamide gels and transferred onto nitrocellulose membranes (Bio-Rad Laboratories Inc., Berkeley, CA, USA). Membranes were blocked in TBST (100 mM Tris-HCL pH 8.0, 150 mM NaCL, 0.5% (*v*/*v*) Tween-20) with low fat-dried milk and then incubated overnight at 4 °C with the appropriate primary antibodies. The specific protein signal was developed using the corresponding secondary antibodies and enhanced chemiluminescence western blotting detection system (ECL TM; Amersham Pharmacia, GE Healthcare), according to the manufacturer´s instructions. Images were acquired for densitometric analysis with a Gel Doc EZ System (Bio-Rad Laboratories Inc., Berkeley, CA, USA), using Image Lab 6.0.1 software (Bio-Rad Laboratories Inc., Berkeley, CA, USA). To normalize protein expression from the same sample and on the same blot, the band intensity of the target protein was divided by the band intensity of the loading protein. 

### 4.5. Immunofluorescence and Confocal Microscopy Analysis

Cells cultured on coverslips were fixed with 4% paraformaldehyde in PBS for 10 min, permeabilized with 0.2% Triton X-100-PBS, blocked with 0.5% fetal bovine serum and 3% bovine serum albumin (BSA) in PBS and then incubated overnight at 4 °C with the corresponding primary antibodies. The following day, cells were washed with 0.05% Triton-X-100-PBS for 5 min and then with PBS alone three times, prior to be incubated for 1 h at room temperature with the appropriate fluorochrome-conjugated secondary antibody. For double immunolabeled samples, this was followed by overnight incubation at 4 °C with corresponding primary antibodies and the next day, cells were incubated with secondary fluorochrome-conjugated antibodies. Where indicated, F-actin was labelled using TRITC-conjugated Phalloidin (Sigma-Aldrich St. Louis, MO, USA) diluted 1:500 in PBS for 10 min at room temperature. Finally, coverslip preparations were incubated for 20 min at room temperature with 0.2 µg/mL diamidino-2-phenylindole (DAPI; Sigma Aldrich) for nuclei visualization, mounted on microscope slides with VectaShield (Vector Laboratories Inc. Burlingame, CA, USA) and further analyzed by confocal laser scanning microscopy (CLSM; Eclipse Ti Series, Nikon Corporation Healthcare Business Unit, Japan) using a 63× (NA = 1.2) oil-immersion objective. The analysis of digitized images was carried out using ImageJ, 1.49 software (Wayne Rasband National Institutes of Health, USA. http://imageJ.nih.gov.ij). For morphometric analysis of nuclei, raw images were calibrated and converted to 8-bit gray scale, to set up a threshold for nuclei selection. Then, nuclear area and circularity parameters were calculated, as described previously [58]. The nucleolar area (μm^2^) was calculated on maxima projection images, using a 3D objects counter, as described previously [59]. To quantify the fluorescence intensity of γ-H2AX (DNA-damage marker and H3K9me3 (heterochromatin marker) foci, the Find Maxima function from ImageJ was used, as previously described [60]. Data were plotted using Prism6 software.

### 4.6. Flow Cytometry and Cell Proliferation Assays

Cells were trypsinized and washed twice with PBS prior to being fixed with 80% ethanol for 30 min, stained for DNA with 1 μg/mL DAPI (Sigma-Aldrich) for 20 min and transferred to flow cytometry tubes for cell cycle analysis in a BD LSR-Fortessa flow cytometer (BD Biosciences, San Jose, CA, USA), using the ModFit LT software (Verity Software House, Topsham, ME). For proliferation assays, cells were harvested and plated in triplicate onto 12-well microplates at (Corning, Costar), at a density of 1 × 10^3^ cells/mL. Cell proliferation was assessed for 10 days using the MTT [3-(4,5-dimethylthiazole)- 2-5-diphenyl tetrazolium bromide] commercial kit (Sigma-Aldrich) and following the manufacturer’s instructions. Absorbance was measured at 570 nm on a Molecular Devices Spectra Max Plus384 microplate reader (Molecular Devices, Sunnyvale, CA, USA).

### 4.7. Fluorescence in Situ Hybridization (FISH) and Relative Telomere Length Determination

Cells grown on coverslips were fixed with 4% paraformaldehyde in PBS 1× for 10 min, washed three times in PBS 1X and permeabilized with Triton 0.2% in PBS 1X for 12 min. Cell preparations were treated for 20 min at 37 °C with 100 µL of RNase (1 μg/mL), washed three times with PBS 1X and dried. Afterwards, coverslips were incubated in hybridization buffer (20 mM Na2HPO4 [Ph 7.4], 20 mM Tris [pH 7.4], 60% formamide, 10% BSA) with 1ng/μL Cy3-conjugated telomere probe (Cy3 conjugated G-strand probe [5´-GGGTTAGGGTTAGGGTTA-3´]) added. Coverslips were then incubated at 80 °C for 2 h in the dark for denaturation, prior to incubation at room temperature overnight for hybridization. Next day, coverslips were washed twice with SSC 2×/1% Tween 20, for 10 min at 60 °C; twice with SSC 1×/0.1% Tween 20 and once with SSC 0.5×/0.1% Tween 20. Finally, cells preparations were incubated for 10 min at room temperature with DAPI (0.2 μg/μL, Sigma-Aldrich Inc.) for nuclei visualization, washed with PBS 1×, and mounted on microscope slides with VectaShield (Vector Laboratories, Inc., Burlingame, CA, USA) for confocal microscopy analysis. The analysis of the number of telomere foci and its relative length of the Q-FISH technique was performed using the Find Maxima function of ImageJ software, 1.49 version image analysis (Wayne Rasband National Institutes of Health, USA. http://imageJ.nih.gov.ij). Raw images were converted to 8-bit gray scale to set up a binary mask that allowed the analysis of fluorescence intensity of the foci within a DAPI-positive region. The relative telomere length was calculated as follows: the telomere mean intensity was divided by the sum intensity of the DAPI signal, as described previously [61].

### 4.8. Senescence-Associated β-Galactosidase (SA-β-Gal) Assay

Cells seeded on coverslips were stained with SA-β-Gal following manufacturer’s instructions (Senescent Cell Histochemical Staining Kit, Sigma-Aldrich, St. Louis, MO, USA). Blue stained cells expressing β-galactosidase (senescent cells) were observed under bright-field microscopy using differential interference contrast.

## 5. Conclusions

In summary, overall our data are consistent with the paradigm that interfering with DG function results somehow in aberrant multipolar mitoses, which in turn evokes a p53-dependent DNA-damage response, arresting the cell cycle progression and thereby inducing senescence, to avoid the propagation of damaged genomes.

## Supplementary Materials

Supplementary materials can be found at https://www.mdpi.com/1422-0067/21/14/4961/s1.

## Abbreviations

NaBu	Sodium butyrate
PM	Plasma membrane
NE	Nuclear envelope
DAPs	Dystrophin-Associated Proteins
ECM	Extracellular matrix
SA-β-gal	Senescence associated β-galactosidase
FACs	Fluorescence-activated cell sorting
SDS PAGE	Sodium dodecyl sulfate polyacrylamide gel
CLSM	Confocal laser scanning microscopy analysis
