# Supplementary figures and images for: Loss of Dystroglycan Drives Cellular Senescence via Defective Mitosis-Mediated Genomic Instability
Source: Int J Mol Sci. 2020 Jul 14;21(14):4961. doi: 10.3390/ijms21144961 (PMC7404207; doi:10.3390/ijms21144961)

A

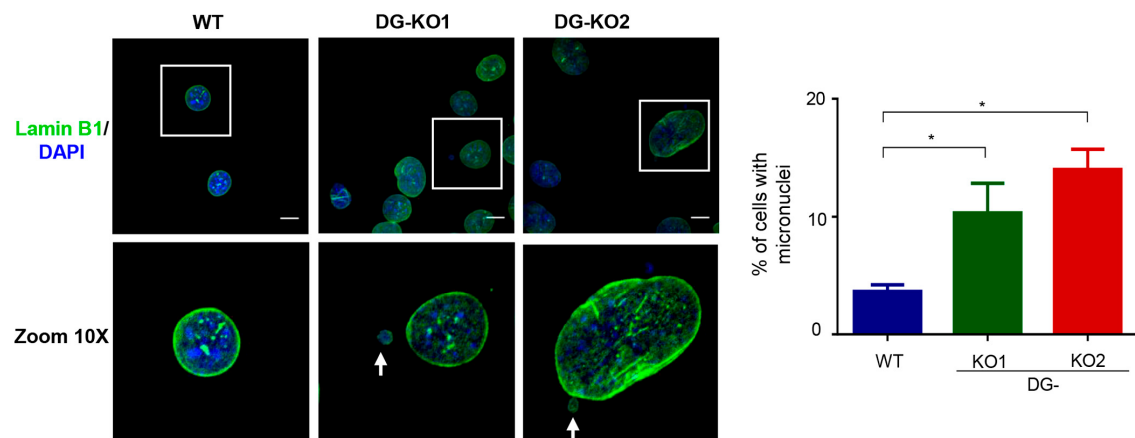

Supplement: Supplementary file 1 [file ijms-21-04961-s001.pdf]
